# Identification of potential diagnostic biomarkers associated with periodontitis by comprehensive bioinformatics analysis

**DOI:** 10.1038/s41598-023-50410-y

**Published:** 2024-01-02

**Authors:** Sixue Gao, Meina Lin, Wei Chen, Xinren Chen, Zhiying Tian, Tong Jia, Yang Xue, Jie Song, Yongping Lu, Linxi Zhou, Liuzhong Wu

**Affiliations:** 1 Department of Periodontics, Shenyang Stomatological Hospital, Shenyang, China; 2 Dalian Medical University, Dalian, China; 3 NHC Key Laboratory of Reproductive Health and Medical Genetics (China Medical University) & Liaoning Key Laboratory of Reproductive Health, Liaoning Research Institute of Family Planning (The Affiliated Reproductive Hospital of China Medical University), Shenyang, China; 4 Department of Orthodontics, Shanghai Ninth People’s Hospital, Shanghai Jiao Tong University School of Medicine, College of Stomatology, Shanghai Jiao Tong University, National Center for Stomatology, National Clinical Research Center for Oral Diseases, Shanghai Key Laboratory of Stomatology, Shanghai, 200125 China

**Keywords:** Computational biology and bioinformatics, Biomarkers, Medical research

## Abstract

Periodontitis is a chronic inflammatory disease that affects the tissues surrounding the teeth, including the gums and the bones supporting the teeth. Early detection and intervention are crucial for effective management of periodontitis. Our study aims to identify a diagnostic biomarker for periodontitis and explore the pathways associated with the occurrence and development of periodontitis. The expression of gingival tissue from periodontitis and healthy control were downloaded from the Gene Expression Omnibus. The weighted gene co-expression network analysis (WGCNA) were used to analyze module genes associated with periodontitis and DESeq2 were performed to identify differently expressed genes (DEGs) between periodontitis and healthy control. Then the candidate genes were obtained by intersecting the genes from interest modules and DEGs. Functional enrichment analysis was performed using gene ontology and kyoto encyclopedia of gene and genomes, followed by the protein–protein interaction (PPI) network analysis. The hub genes were identified by the cytoCNA plugin in Cytoscape. Finally, immunohistochemical staining of the hub genes was performed to validate the findings. WGCNA analysis found that the expression of the MEblack module was significantly higher in individuals with periodontitis compared to those in the healthy control group. A total of 888 DEGs, including 750 upregulated and 138 downregulated genes, were identified. Finally, 427 candidate genes were identified potentially associated with periodontitis after intersecting the DEGs and the black module genes. Several critical signaling pathways were identified associated with periodontitis by functional enrichment analysis, including cytokine–cytokine receptor interaction, neutrophil extracellular trap formation, *Staphylococcus aureus* infection, and Interleukin-17 signaling pathway. The PPI network analysis revealed that C-X-C motif chemokine ligand 5 (CXCL5) and C-X-C motif chemokine ligand 6 (CXCL6) could play an important role in the process of periodontitis. The gene expression level of CXCL5 and CXCL6 detected using immunohistochemical verified the findings. In conclusion, we found that CXCL5 and CXCL6 are closely associated with the occurrence of periodontitis. Our present pilot study suggests that CXCL5 and CXCL6 have the potential to be used as a diagnostic biomarker of periodontitis.

## Introduction

Periodontitis is a chronic inflammatory condition that affects the tissues surrounding the teeth. If not properly treated, periodontitis can lead to the destruction of the gums, periodontal ligaments, and alveolar bone that support the teeth. This can cause symptoms such as gum inflammation, bleeding, halitosis, and eventually tooth loss. In addition to its impact on oral health, periodontitis has also been linked with various systemic conditions, including diabetes, cardiovascular diseases, respiratory diseases, Alzheimer's disease, and adverse pregnancy outcomes^1,2^.

Periodontitis is caused by the complex interaction between periodontal microorganisms and their metabolic products, which could trigger an inflammatory response in the host. This process is initiated when a biofilm forms near the gums and releases various substances, such as lipopolysaccharides, peptidoglycans, and toxins, leading to the host's responses^3^. Inflammation of the periodontal connective tissue leads to the loss of attachment between the bone and teeth, and epithelial cell proliferation along the surface of the tooth root, resulting in the formation of a deep pocket. As the periodontal pocket deepens, the extent of inflammatory infiltration also increases^4^. The diagnosis of this disease largely relies on clinical and radiographic examinations, such as probing depth, bleeding on probing, clinical attachment level, and radiographic assessment of alveolar bone volume^5^. Similar to other chronic diseases in humans, there are still significant challenges in the diagnosis and classification of different forms of periodontal disease^6^.

Biomarkers are biological indicators associated with the occurrence or progression of pathology^7^, which can be measured accurately and rapidly and share high prognostic and predictive value. In short, markers of disease presence or progression are determined to be able to predict the progression of the disease and markers of treatment response are to be able to indicate the most appropriate indications for medication and response types. Currently, some biomarkers such as interleukins, growth factors, complement components, and other biomolecules have been proven useful in the diagnosis and follow-up of periodontitis, aiding in the early assessment and monitoring of disease progression and treatment response^7,8^.

Chemokines are a type of cell factors that could induce specific cells to migrate towards inflammatory tissues and play a crucial role in regulating the immune response. Any abnormalities in the chemokine signaling pathway can potentially have detrimental effects, leading to the development of inflammatory diseases and cancer^9^. C-X-C motif chemokine ligand 5 (CXCL5) and C-X-C motif chemokine ligand 6 (CXCL6) are types of chemokines that could participate in the occurrence of various inflammatory diseases through their immunoregulatory amplification effects. Some studies have shown that early inflammatory events, including leukocytosis and the production of acute-phase reactants, could lead to increased production of chemokines^10^. Several researches have demonstrated that chemokines could play a regulatory role in periodontal disease and bone remodeling under physiological and pathological conditions^11^. However, the relationship between CXCL5 and CXCL6 and periodontitis is not yet fully understood.

In this study, our objective was to identify a diagnostic biomarker for periodontitis by analyzing the gene expression levels of periodontal tissues from patients with periodontitis and healthy controls using bioinformatics methods. In addition, we aimed to discuss the pathways associated with the occurrence and development of periodontitis and validate the protein expression levels using immunohistochemistry. Our study might offer a new perspective on the diagnostic biomarkers for periodontitis.

## Material and methods

### Data collection

**Figure 1:**
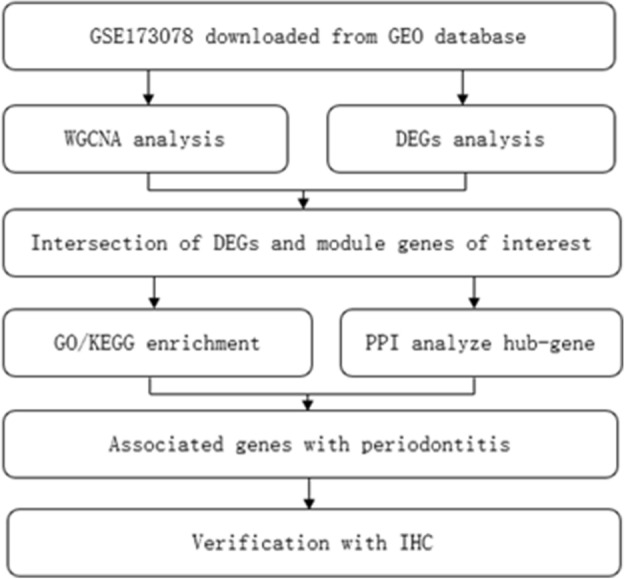
The analysis flow chart used in this study.

The mRNA transcription of gingival tissues was downloaded from the Gene Expression Omnibus (GEO) database (http://www.ncbi.nlm.nih.gov/geo). The inclusion criteria of gene expression profiles are as follows: (1) mRNA expression profiling by high throughput sequencing; (2) the sample size is larger than 6. Datasets with other treatments, such as drug, genome editing or vehicle administration were excluded. As a result, the datasets GSE173078, consists of 12 periodontitis, 12 gingivitis (not included in the subsequent analyses), and 12 healthy controls transcriptome sequencing results, were selected to identify biomarkers of periodontitis in this study. The analysis flow chart of this study was presented in Fig. 1.

### WGCNA construction and differentially expressed genes analysis

The weighted gene co-expression network analysis (WGCNA) package in R software was used to construct a gene co-expression network. The correlation matrix and topological overlap matrix between genes were constructed to measure the network connectivity of genes. Genes with similar expression levels were divided into different modules. And then, the weight of each module in the dataset was calculated and the dataset with the highest weight was selected. The minimal module size, cut height and soft-thresholding power were set at 60, 0.25 and 9, respectively. The modules with biggest R value were selected as interest gene modules.

The differentially expressed genes (DEGs) between periodontitis patients and healthy controls were conducted by R package DESeq2. A criterion of |log2(FoldChange)|> 1 and a false discovery rate (FDR) < 0.05 were used to identify significant genes. Then the candidate genes were obtained by intersecting the genes from interest modules and DEGs.

### Functional enrichment analysis

To further clarify the role of candidate genes in the occurrence of periodontitis, gene ontology (GO) enrichment and kyoto encyclopedia of gene and genemos (KEGG) analysis were performed by R package “clusterprofiler”. The R package “org.Hs.eg.db” were used to convert the gene names to gene ID.

### Identification and validation of hub genes

The protein–protein interaction (PPI) network of the candidate genes was constructed using the Search Tool for the Retrieval of Interacting Genes (STRING) database (https://string-db.org/). The interactions of candidate genes were visualized using Cytoscape 3.7.1 (https://www.bytesin.com/software/Cytoscape/). The hub genes were identified by the “cytoNCA” plugin. Genes with the highest degree centrality (DC), between centrality (BC), closeness centrality (CC) were considered as the hub genes. Then, we utilized the R package “pROC” to calculate the receiver operating characteristic (ROC) curve and the area under the ROC curve (AUROC) to evaluate the diagnostic potential of hub genes.

### Patient recruitment and immunohistochemical verification

To evaluate the diagnostic value of the candidate genes, we conducted Immunohistochemical analysis on the periodontal tissues of 12 patients with periodontitis as well as healthy controls. According to the criteria established by the American Academy of Periodontology (AAP) and the European Federation of Periodontology (EFP) in 2017^12^, the diagnosis of periodontitis was made if either of the following criteria is met: the presence of ≥ 2 non-adjacent teeth with clinical attachment loss (CAL) on interproximal surfaces; or the presence of ≥ 2 teeth with buccal or lingual CAL ≥ 3 mm, along with periodontal pockets ≥ 3 mm. On the contrary, the inclusion criteria for healthy controls in this study were: (a) probing depth of ≤ 3 mm and no attachment loss in full-mouth periodontal examination; (b) no more than 10% of sites with bleeding index ≥ 2; (c) gender and age matched with the case group. The exclusion criteria were: (a) history of systemic diseases such as cardiovascular disease, autoimmune or inflammatory diseases; (b) patients with malignant tumors; (c) pregnant or lactating women. The study protocol was reviewed and approved by the Ethics Committee of Shenyang Stomatological Hospital. Written informed consent was obtained from all participants before collecting periodontal tissue samples. All methods were performed in accordance with the relevant guidelines and regulations. Immunohistochemical analysis of the candidate genes was performed following the instructions provided in the kit (E-IR-R217, Elabscience Biotechnology Co., Ltd Wuhan, China). The protein expression levels in the Immunohistochemistry (IHC) sections were assessed by measuring the relative integrated optical density (IOD) using ImageJ software (National Institutes of Health, Bethesda, MD, USA).

### Statistical analysis

The candidate gene analysis and the generation of figure were performed using R studio (version 4.0.4). The IOD data of IHC was analyzed by the Student’s t-test and the image was generated by GraphPad Prism 7 software (GraphPad Software, Inc., La Jolla, CA, USA).

### Ethics approval and consent to participate

The study protocol was reviewed and approved by the Ethics Committee of Shenyang Stomatological Hospital.

## Results

### WGCNA and module analysis

**Figure 2:**
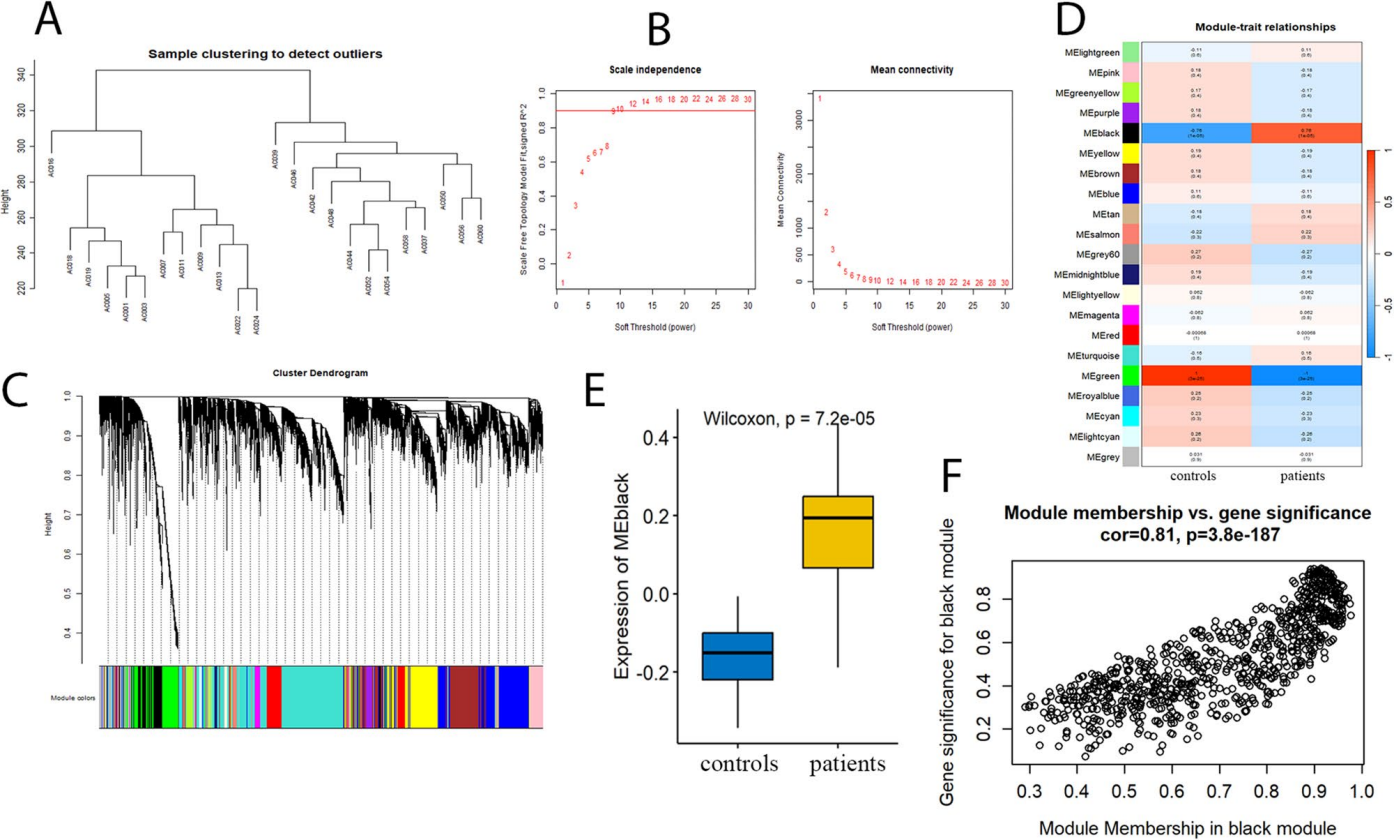
WGCNA and module analysis. (**A**) Clustering dendrograms of all samples. (**B**) Scale-free fitness index and mean connectivity for features with different soft-thresholding powers. (**C**) Clustering dendrogram of filtered genes based on a dissimilarity measure of topological overlap matrix. (**D**) Heatmap of the correlation between module eigengenes and periodontitis. (**E**) The expression of the MEblack module in periodontitis patients verses healthy control. (**F**) Scatter plot of the correlations between the black module genes and periodontitis.

In this study, median absolute deviation was used to filter the expression data of all genes, and a total of 13,951 genes (top 75%) were finally retained. Hierarchical clustering was used to detect outliers and remove the abnormal samples. All samples were well divided into two groups and there is no outlier data (Fig. 2A). The soft thresholding power β = 9 was determined using the pickSoftThreshold function in the WGCNA package based on the scale-free network evaluation coefficient R^2^ reaching 0.9 at first time (Fig. 2B). A cut height of 0.25 was used to merge modules with similar expressions (Fig. 2C). Finally, all genes were divided into 21 different modules (Fig. 2D). Among them, the MEblack module, consisting of 800 genes, showed the highest positive module-trait association (r = 0.76, *p* < 0.001), while the MEgreen module, consisting of 958 genes, showed the highest negative module-trait association (r = − 1, *p* < 0.001). The expression of the MEblack module was found to be significantly higher in individuals with periodontitis compared to those in the healthy control group (Fig. 2E). The module membership versus gene significance in the MEblack module was shown with a scatter diagram (Fig. 2F).

### Identification of DEGs and candidate genes

**Figure 3:**
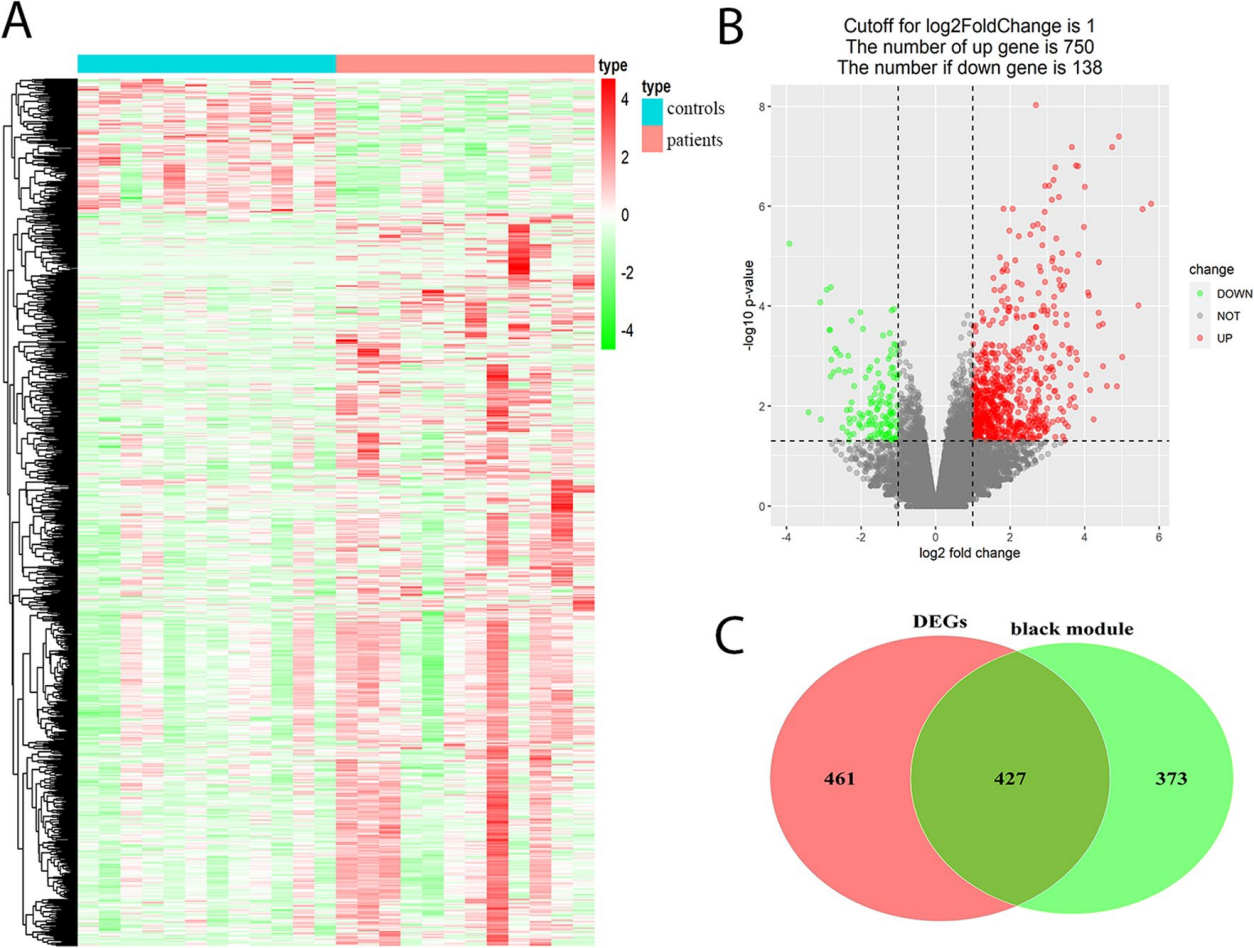
Identification of DEGs and candidate genes. (**A**) The heatmap of DEGs in periodontitis patients and health control. (**B**) Volcano map of the DEGs identified by DEseq2 packge. (**C**) Venn diagram of the intersection of DEGs and black module genes.

DESeq2 package was used to calculate gene expression differences between periodontitis patients and healthy controls. A total of 888 DEGs, including 750 upregulated and 138 downregulated genes, were identified (Fig. 3A,B). After intersecting the DEGs and the black module genes which highly expression in periodontitis patients, we finally identified 427 candidate genes that were potentially associated with periodontitis (Fig. 3C).

### Functional enrichment and identification of hub genes

**Figure 4:**
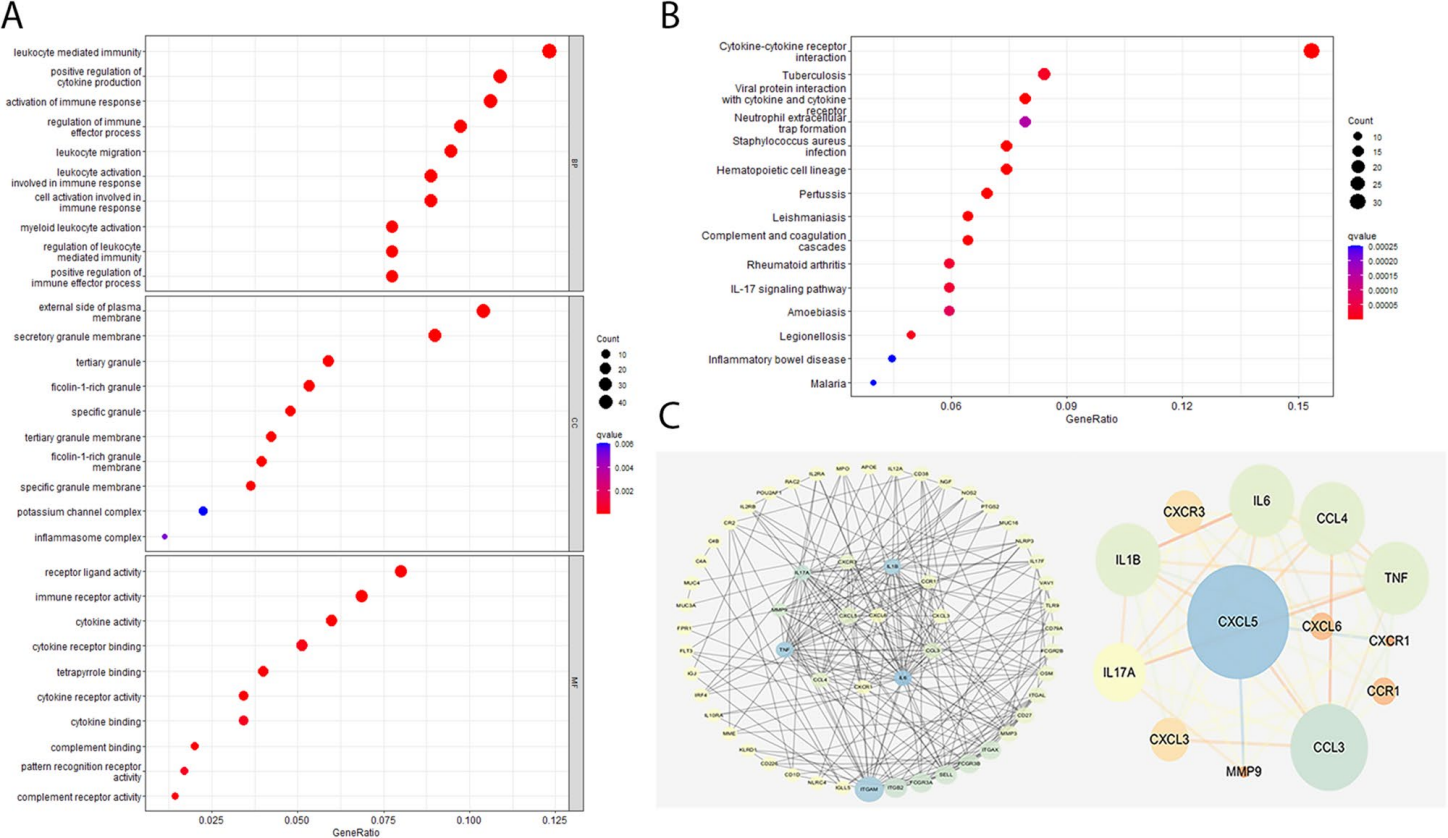
Functional enrichment and identification of hub genes. (**A**) Top 10 enriched GO terms of candidate genes. (**B**) Top 15 enriched KEGG terms of candidate genes. (**C**) PPI network illustrated by Cytoscape.

GO functional enrichment and KEGG pathway analysis were conducted on 427 candidate genes obtained from the intersection of WGCNA and differential genes to investigate their biological functions. With regard to biological processes (BPs) of GO annotation enrichment terms, the candidate genes mainly enriched in leukocyte mediated immunity, positive regulation of cytokine production, activation of immune response, regulation of immune effector process, and positive regulation of immune effector process. As for cell components (CCs), the candidate genes substantially enriched in external side of plasma membrane, secretory granule membrane, and inflammasome complex. For molecular functions (MFs), candidate genes mainly enriched in receptor ligand activity, immune receptor activity, cytokine activity and cytokine receptor activity (Fig. 4A). KEGG pathway analysis identified several critical signaling pathways associated with periodontitis, including cytokine–cytokine receptor interaction, neutrophil extracellular trap formation, *Staphylococcus aureus* infection, and IL-17 signaling pathway (Fig. 4B).

**Figure 5:**
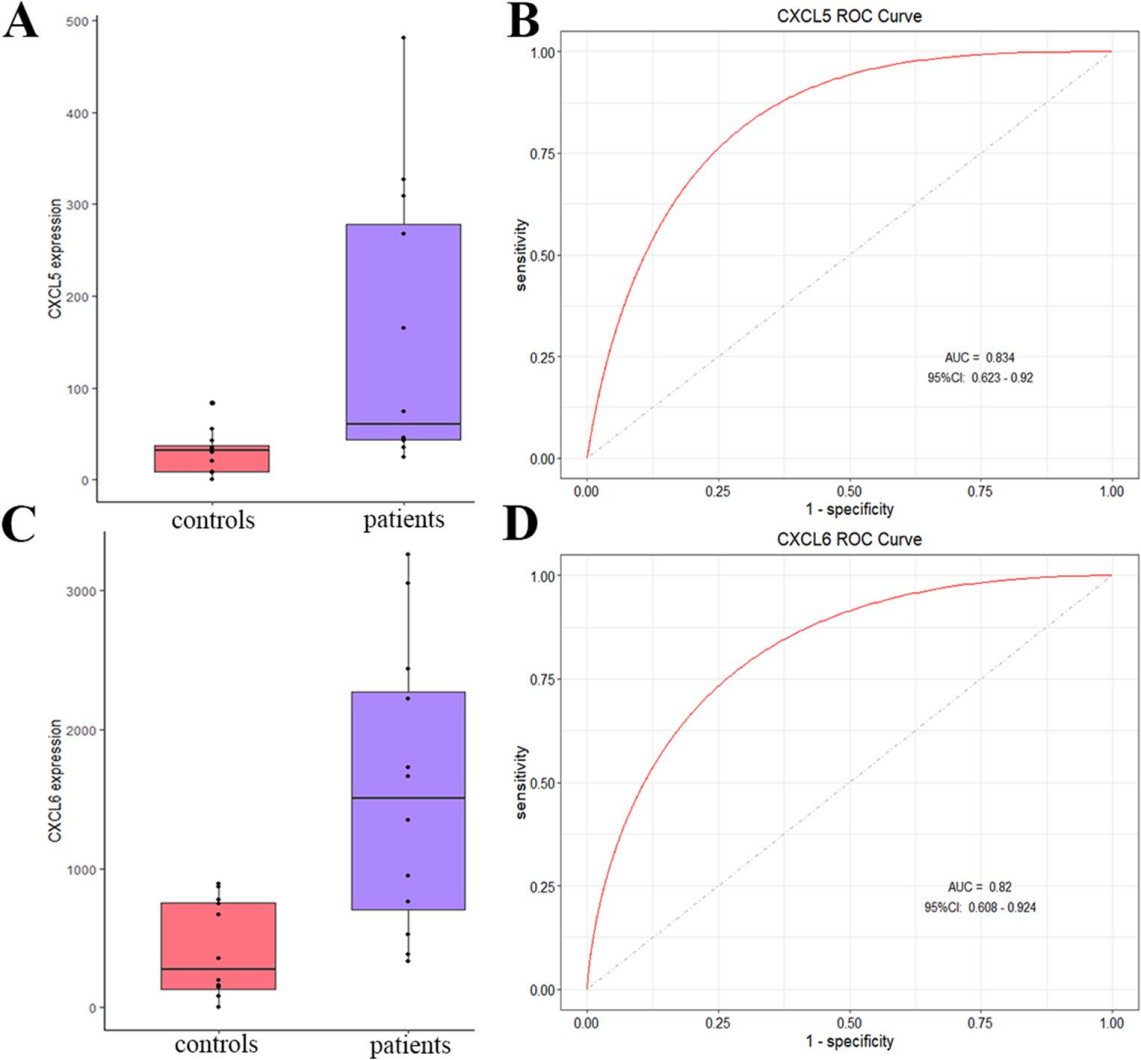
The relative expression and the ROC curve of CXCL5 and CXCL6 genes between periodontitis and controls.

The PPI network of candidate genes was constructed using STRING and cytoscape software (Fig. 4C). Based on the score of DC, BC, and CC in the PPI network, all candidate genes were sorted. The CXCL5 and CXCL6, which were also included in the Interleukin-17 (IL-17) signaling pathway and cytokine-cytokine receptor interaction pathway, have a high score. The expression levels of CXCL5 and CXCL6 were significantly higher in periodontitis patients compared to those in controls (Fig. 5A,C). ROC curve analysis showed that the AUROC values for CXCL5 and CXCL6 were 0.834 and 0.82 (Fig. 5B,D), respectively, indicating that they might have excellent specificity and sensitivity for the diagnosis of periodontitis.

### Gene expression validation by immunohistochemical

**Figure 6:**
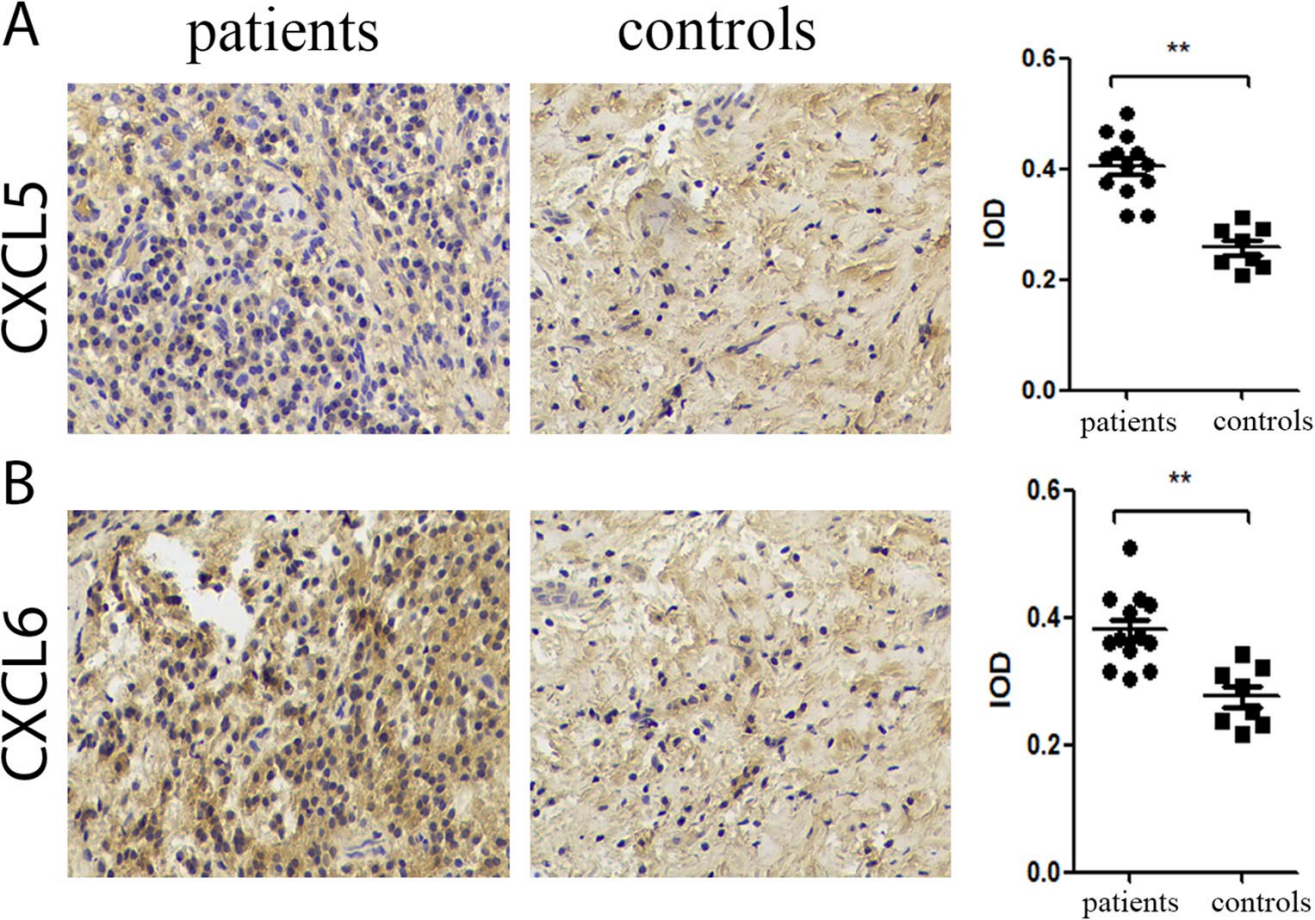
Expression of CXCL5 and CXCL6 genes in periodontitis and health controls. Magnification, × 200, **p < 0.01.

We compared the gene expression level using immunohistochemical, and found that the IOD/Area score of CXCL5 and CXCL6 in periodontitis patients were significantly higher than those in health controls (*p* < 0.01)(Fig. 6A,B).

## Discussion

It is crucial to reveal the regulation of the inflammatory response in order to elucidate the pathogenesis of periodontitis^13^. Despite the rapid progress in the research of the mechanism of periodontitis, the biomarkers for the diagnosis of the disease are still unclear. To preliminarily probe the biomarker diagnosis for diagnosing periodontitis, we performed differential expression analysis and WGCNA analysis on periodontal tissues from 12 patients with periodontitis and healthy controls. By taking the intersection of the results from both analyses, we identified candidate genes associated with periodontitis. The results of the GO enrichment analysis and KEGG pathway enrichment analysis on candidate genes indicated that the candidate genes are mainly enriched in immune receptor activity, cytokine activity, cytokine receptor activity, as well as cytokine-cytokine receptor interaction and IL-17 signaling pathway. The results of the PPI analysis indicated that CXCL5 and CXCL6 are key genes involved in the development of periodontitis.

CXCL5 is a member of the CXC chemokine family and is predominantly expressed in neutrophils and monocytes, with potential expression in eosinophils as well. CXCL5 plays a crucial regulatory role in the inflammation process. Firstly, CXCL5 can promote the infiltration and aggregation of inflammatory cells by chemotaxis of neutrophils and monocytes. Studies have shown that the expression level of CXCL5 is significantly upregulated in various inflammatory diseases, such as rheumatoid arthritis, inflammatory bowel disease, kidney diseases and liver ischemia–reperfusion injury^14–17^. Secondly, CXCL5 also participates in cell interactions and signal transmission during inflammation. Zhang and his colleagues discovered that upon binding to its receptor CXCR2, CXCL5 can activate multiple signaling pathways, including MAPK, NF-κB, and STAT3. This activation subsequently regulates the activation, proliferation, and secretion of inflammatory mediators by inflammatory cells^18^. CXCL5 can also regulate the polarization of macrophages and the production of cytokines, thereby affecting the development and progression of inflammation^19^. In this study, we found a significant correlation between the CXCL5 gene and the occurrence of periodontitis through differential expression analysis and WGCNA enrichment analysis. The functional enrichment results showed that CXCL5 is involved in the IL-17 signaling pathway, which is closely related to the occurrence of periodontitis. The result of cytoCNA plugin in Cytoscape also showed that CXCL5 is the hub of candidate genes. To confirm our findings, we analyzed the expression level of the CXCL5 gene in periodontal tissues of 12 patients with periodontitis and healthy controls using immunohistochemistry. The results were consistent with the bioinformatics analysis, showing a significant increase in the expression level of the CXCL5 gene in patients with periodontitis.

CXCL6 is a chemokine factor with a conserved Glu-Leu-Arg (ELR) motif^20^. Similar to CXCL5, it exhibits potent chemotactic properties and promotes angiogenesis effectively. Papapanou1 et al. analyzed the mRNA level of periodontal tissues from 184 periodontitis patients and 63 healthy control using microarray chips and found that the expression levels of most chemokine families were elevated, with cxcl6 showing the highest expression^21^. CXCL6 also plays an important role in diabetic nephropathy (DN). Studies have reported that CXCL16/CXCL6 can promote lipid accumulation in renal tubular epithelial cells, thereby accelerating tubular injury^22^. Additionally, CXCL6 can accelerate the development of renal fibrosis through the JAK/STAT3 signaling pathway^23^. Both the in-silico analysis and the experimental results showed that CXCl6 is involved in the development of periodontitis.

Numerous studies have reported potential biomarkers associated with predicting periodontitis, such as MMP-8^24,25^ and IL-8^21^. Lin Zhang et al.^25^ carried out a meta-analysis studying the correlation between MMP-8 and periodontitis. They found that in eight studies, the levels of MMP-8 in the saliva of periodontitis patients were significantly higher compared to healthy individuals. However, two studies reported no significant difference. Due to the high heterogeneity and publication bias, further studies are needed to identify the correlation between MMP-8 and periodontitis. IL-8 is continuously expressed in periodontal tissues and mediates the recruitment of neutrophils to gingival tissues^26^. Studies have reported that IL-8 expression is significantly increased in periodontal disease and is associated with the occurrence of periodontitis^27,28^. Moritz et al. demonstrated that as the severity of periodontal lesions increases, neutrophil recruitment mediated by CXCL6 can supplement the recruitment pathway of IL-8^21^. Currently, there is limited research on CXCL5 and CXCL6 as biomarkers for periodontitis. Our study suggests that they might have predictive potential, and combining CXCL5 and CXCL6 with other biomarkers may enhance the ability to predict periodontitis. Our study has indeed several limitations that should be acknowledged. Firstly, the sample size of human subjects was limited, and therefore, further studies with larger sample sizes are necessary to validate our findings. Secondly, we solely relied on immunohistochemistry techniques to validate the hub genes in vitro. It would be beneficial to conduct additional experiments using more quantitative methodologies to further demonstrate our results. Lastly, the exact role of the identified hub genes in periodontitis requires further elucidation, both in vitro and in vivo. Further research is crucial to determine whether CXCL5 and CXCL6 can serve as predictive biomarkers for the diagnosis and treatment of periodontitis.

## Conclusion

In conclusion, we integrated multiple bioinformatics analyses and found that CXCL5 and CXCL6 are closely associated with the occurrence of periodontitis. Immunohistochemistry experiments further confirmed the important role of cxcl5 in the pathogenesis of periodontitis. Our present pilot study suggests that CXCL5 and CXCL6 have the potential to be used as a diagnostic biomarker of periodontitis.

## Data Availability

The datasets analysed during the current study are available from the GSE173078 of GEO database.
